# Effects of selenium-enriched yeast dietary supplementation on egg quality, gut morphology and caecal microflora of laying hens

**DOI:** 10.1080/10495398.2023.2258188

**Published:** 2024-01-09

**Authors:** Ruili Li, Jiewei Liu, Minxiao Liu, Mingzhi Liang, Zengguang Wang, Yufen Sha, Huiwen Ma, Yafeng Lin, Baohua Li, Jinming You, Lei Zhang, Ming Qin

**Affiliations:** a Institute of Animal Science and Veterinary Medicine, Yantai Academy of Agricultural Sciences, Yantai, China; b College of Animal Science and Technology, Jiangxi Agriculture University, Nanchang, China; c Yantai Animal Disease Prevention and Control Center, Yantai, China; d Yantai Agricultural Technology Extension Center, Yantai, China; e Haiyang Animal Disease Prevention and Control Center, Yantai, China

**Keywords:** Gut microbiota, laying hens, feeding supplements, selenium-enriched yeast

## Abstract

Selenium (Se) is an essential micronutrient for humans and animals and is a powerful antioxidant that can promote reproductive and immune functions. The purpose of this study was to evaluate the effects of supplemental dietary selenium-enriched yeast (SeY) on egg quality, gut morphology and microflora in laying hens. In total, 100 HY-Line Brown laying hens (45-week old) were randomly allocated to two groups with 10 replicates and fed either a basal diet (without Se supplementation) or a basal diet containing 0.2 mg/kg Se in the form of SeY for 8 weeks. The Se supplementation did not have a significant effect on egg quality and intestinal morphology of laying hens. Based on the 16S rRNA sequencing, SeY dietary supplementation effectively modulated the cecal microbiota structure. An alpha diversity analysis demonstrated that birds fed 100 mg/kg SeY had a higher cecal bacterial diversity. SeY dietary addition elevated *Erysipelotrichia* (class), *Lachnospiraceae* (family), *Erysipelotrichaceae* (family) and *Ruminococcus_torques_group* (genus; *p* < .05). Analysis of microbial community-level phenotypes revealed that SeY supplementation decreased the microorganism abundance of facultatively anaerobic and potentially pathogenic phenotypes. Overall, SeY supplementation cannot significantly improve intestinal morphology; however, it modulated the composition of cecal microbiota toward a healthier gut.

## Introduction

Animal diets contain both inorganic and organic sources of selenium (Se).^1^ There have been numerous researches on organic Se from selenium-enriched yeast (SeY) in animal nutrition over the years.^2–5^ In addition, the effects of organic Se sources, such as selenomethionine,^6–9^ 2-hydroxy-4-methyselenobutanoic acid^10–12^ and Se-enriched alga,^13^^,^^14^ as well as Se-enriched probiotics,^15^ have also been studied in domestic animals and poultry. In comparison to inorganic Se sources, SeY offers significant benefits as a feed supplement.

Se plays a significant role in antioxidation, antistress, immune function and reproduction performance by being a component of several antioxidant enzymes and selenoproteins.^16^ Furthermore, Se deficiency can also cause human and animal diseases, such as Kaschin–Beck in humans; exudative quality and pancreatic trophic atrophy in poultry;^17^^,^^18^ and white muscle disease in sheep.^19^ Thus, foods and feeds containing Se are crucial for preventing diseases associated with Se deficiency.

SeY is an organic Se source with high Se content, converted by microorganisms.^20^ Compared with inorganic Se, organic Se has higher bioavailability and retention in broilers.^21^^,^^22^ A diet supplemented with Se will affect the selenium content of eggs and SeY has a higher deposition efficiency than inorganic Se.^23^ SeY can significantly increase the daily feed intake and eggshell strength of laying hens when compared with inorganic Se.^24^ It may be that SeY improves a chicken’s redox state, thereby reducing the oxidative stress they experience as a result of high temperature and enteric bacterial infection.^25^ More importantly, SeY has a range of advantages over inorganic Se, including strong activity, low toxicity, high absorption rate and low environmental pollution.^26^^,^^27^ In order to improve chicken production performance, it is crucial to use SeY in a reasonable manner.

Currently, the majority of studies examine the effects of supplementing laying hens with selenium at different levels on their performance.^15^^,^^24^^,^^28^ The pattern of host–microbe interactions drives the genetic and phenotypic diversities of gut microbiota to affect the physiological, immunological and nutritional status of the host. There are problems with production performance, immunity and egg quality decline and tissue and organ damage of layers at the late laying stage. However, the potential effect of SeY on egg production of late-phase laying hens is still ambiguous, and the associations between dietary SeY, laying performance and the microbiota require further elucidation. The effect of adding Se to laying hen feed on their intestinal health is not well studied. It was the objective of this study to investigate the effects of SeY on egg quality, gut morphology and cecal microflora population, and to provide a theoretical basis for its application in the production practice of laying hens.

## Materials and methods

### Ethics statement

All animal care and treatment procedures were approved by the Animal Ethics Committee of Shandong Agricultural University, China and performed following the Committee’s guidelines and regulations (Approval No.: 2004006).

### Preparation of SeY

The SeY was purchased from Hubei Angel Yeast Co., Ltd, which contains 2000 mg/kg Se. The dose of dietary SeY supplement was added according to the previous pretest results and the reference to the commercial recommendation of company.

### Experimental birds, diet and management

A total of 100 HY-Line Brown commercial laying hens at 45 wk of age were randomly assigned to two groups with 10 replicates of 5 birds each (50 laying hens per group). In the early stage, the research team conducted an experiment on the effect of adding different levels of selenium on the selenium content in eggs. The results showed that the selenium content in eggs after adding 100 mg/kg selenium-enriched yeast did not exceed 0.6 at 56 d, while the selenium content in eggs of higher dose groups was higher than 0.6 mg/kg. Therefore, from the perspective of biological safety, the laying hens in this experiment were fed a corn-soybean meal-based diet (Table 1) supplemented with SeY at 0 (control group, Ctrl) and 100 mg/kg. Based on National Research Council (1994) guidelines,^29^ the basal diet was developed. Diets and water were provided ad libitum in mash form and by nipple drinkers, respectively. During the 8-week experiment period, artificial light was offered by a 16-h light and an 8-h dark cycle, and the temperature varied between 22 and 25 °C.

**Table 1.** Dietary composition and nutrient levels of the experimental diet (as fed basis).

Item, %	Proportion
Corn	63.50
Soybean meal	24.50
Soybean oil	1.20
Shell powder	4.60
Stone powder	4.15
Salt	0.30
Mono-dicalcium phosphate (MDCP)	0.75
Premix	1.0
Total	100.00
Nutrient content, %	
Crude protein	16.41
Lysine	0.89
Methionine	0.65
Methionine + Cysteine (M + C)	0.47
Metabolizable energy, MJ/kg	11.56
Calcium	3.20
Total phosphorous	0.56
Available phosphorous	0.28

*Note*: Each kg of premix contains: vitamin A108000IU, vitamin D3 3000 IU, vitamin E 20 IU, vitamin K 32 mg, vitamin B1 0.4 mg, vitamin B2 3.0 mg, vitamin B6 1.0 mg, vitamin B12 0.006 mg, biotin 0.05 mg, pantothenic acid 12 mg, folic acid 0.1 mg, nicotinic acid 7.0 mg, Iron 80 mg, Manganese 100 mg, Zinc 75 mg, Iodine 0.8 mg, Selenium 0.35 mg.

### Sample collection and analytical determination

Thirty eggs (5 eggs/replicate) were randomly collected on day 28, 42 and 56 of the experiment to determine the egg quality. Individual eggs were weighed with a digital laboratory balance to an accuracy of 0.1 g. An electronic caliper MITUTOYO Absolute Digmatic Caliper model CD-15DCX (Japan) exact to 0.01 mm was used to measure the long and short diameters of eggs, and the ratio of short diameter to long diameter was measured using an egg shape index.

After Se supplementation for 8 weeks, 5 randomly chosen laying hens from each dietary treatment were slaughtered by cervical dislocation, and the cecum was immediately fixed in 10% phosphate-buffered formalin. Following embedding of the cecal tissues in paraffin, 5 micrometer sections of paraffin were cut and mounted on glass slides. After dewaxing with xylene and hydrating, the sections were recolored with hematoxylin and eosin. Using a light microscope coupled with image processing software (Image J 1.8.0), three complete villi-crypt units were chosen for morphology observation for each sample. The cecal contents were carefully collected and stored at −80 °C until DNA extraction. Total genomic DNA was extracted from cecum contents utilizing QIAamp 96 PowerFecal QIAcube HT kit (QIAGEN, 51531) according to the manufacturer’s protocols. The DNA concentration was confirmed with NanoDrop, and the quality of DNA was evaluated by 1% agarose gels. The 16S rDNA V3-V4 region of the ribosomal RNA gene was amplified by PCR (95 °C for 2 min, followed by 27 cycles at 98 °C for 10 s, 62 °C for 30 s, 68 °C for 30 s and a final extension at 68 °C for 10 min using primers 341 F: CCTACGGGNGGCWGCAG; 806 R: GGACTACHVGGGTATCTAAT). The amplicons were extracted from 2% agarose gels, purified with AxyPrep DNA Gel Extraction Kit (Axygen Biosciences, Union City, CA, USA) according to the manufacturer’s guidelines, and quantified with QuantiFluor-ST (Promega, USA). Purified amplicons were subsequently sequenced using the Illumina MiSeq PE250 platform and bioinformatics analysis was conducted by Genedenove Biotechnology (Co. Ltd. Guangzhou, China).

The raw fastq files were filtered by QIIME (V1.9.0, http://qiime. org/index.html). FLSAH (V1.2.11, http://ccb.jhu.edu/software/FLASH) was used to merge the paired-end reads. The tags were compared to the Gold database utilizing the UCHIME algorithm (http://www.drive5.com/usearch/manual/uchime algo.html) to identify chimeric sequences. The effective tags were clustered and classified into operational taxonomic units (OTUs) of ≥97% similarity by utilizing UPARSE software (V7.0.1001, http;//drive55.com/uparse/). The representative sequences were then assigned the taxonomic category using the SILVA database.

### Statistical analysis

For 16S sequencing data, the alpha and beta diversity analysis was evaluated by QIIME (V1.9.0). The alpha and beta index was compared across groups with Welch’s *t* and Anosim test using R software (V3.4.3), respectively. The data are represented as mean ± standard deviations. Statistical testing was performed using one-way analysis of variance (ANOVA). The means were compared by utilizing *T* test as contained in SAS 9.2 software (SAS Institute Inc., USA). Statistical significance of differences was expressed by the presence of a–b letters within a column; different letters within a column signify a significant difference between means (*p* < .05).

## Results

### Egg quality

The egg quality-related characteristics are showed in Table 2. There was no significant difference in any of the egg quality traits among dietary groups (*p >* .05).

**Table 2.** Effect of SeY on egg quality of laying hens.

Item	Ctrl	SeY	*p* Value
D28			
Egg weight/g	60.53	58.37	.23
Egg long diameter/mm	57.38	56.30	.35
Egg short diameter/mm	43.66	43.21	.31
Egg shape index	0.76	0.77	.75
D42			
Egg weight/g	58.24	56.42	.11
Egg long diameter/mm	56.86	55.89	.10
Egg short diameter/mm	43.37	43.17	.71
Egg shape index	0.76	0.77	.37
D56			
Egg weight/g	59.42	58.66	.45
Egg long diameter/mm	55.83	56.09	.26
Egg short diameter/mm	43.62	43.19	.34
Egg shape index	0.78	0.77	.25

### Morphology of cecum

**Figure 1.**
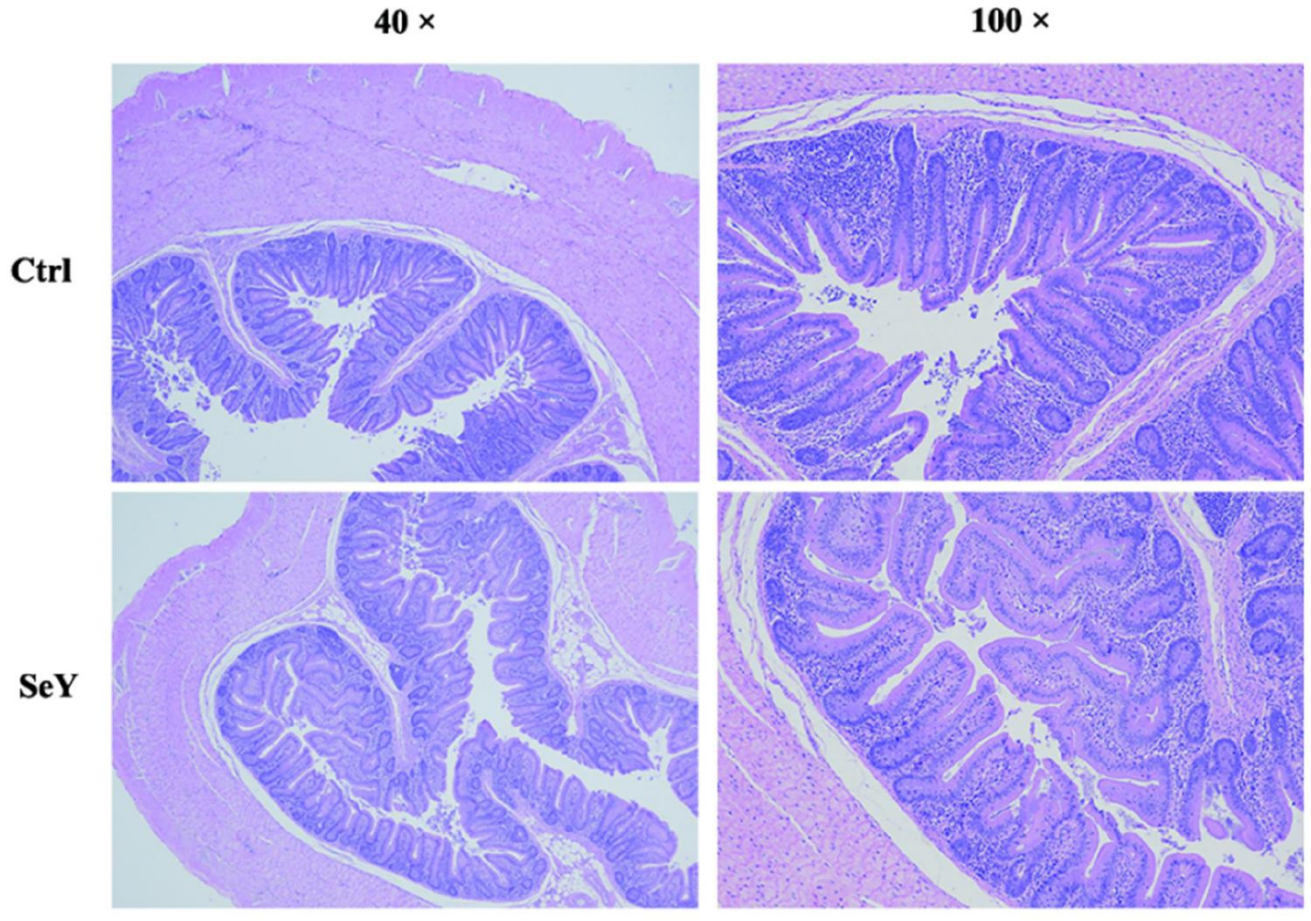
Morphology of cecum of laying hens fed SeY diets. Hematoxylin and eosion (H&E) stating, 40 × and 100 × magnification. Ctrl, basal diet; SeY = supplementation at 100 mg/kg.

Villus height (VH), crypt depth (CD) and villus height/crypt depth (VH/CD) are showed in Fig. 1 and Table 3 to investigate the effects of SeY on cecal morphological features. Compare to controls, hens fed diets added SeY showed no differences in cecal morphology (*p >* .05).

**Table 3.** Effect of dietary SeY supplementation on caecal morphology of laying hens.

Intestinal segment	Item	Ctrl	SeY	*P* Value
Cecum	Villus height, μm	206.66 ± 15.97	224.62 ± 44.81	.72
	Crypt depth, μm	132.57 ± 14.78	113.63 ± 13.91	.38
	Villus/Crypt ratio	1.65 ± 0.22	2.09 ± 0.46	.41

Five replicates per treatment.

### Generation of OTUs

**Figure 2.**
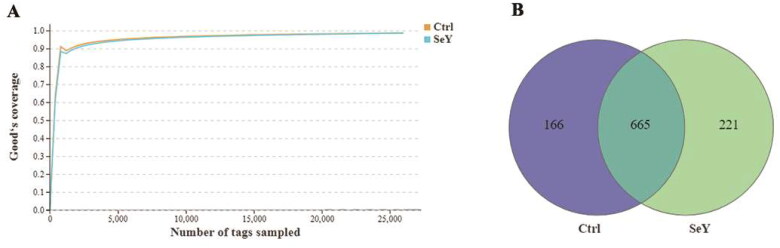
Numbers of the cecal OTUs in the two dietary groups. (A) Rarefaction curves of good’s coverage reached saturation in different groups. (B) Venn diagram of OTUs of gut microbiota in different groups of laying hens.

The microbiome modulations in layer cecum after SeY supplementation were identified by targeting the V3–V4 region of 16S rDNA utilizing the Illumina MiSeq platform. The rarefaction curves for each group were infinitely close to the saturation plateau, proving that the sequencing results contained enough depth to capture most microbial diversity information (Fig. 2A). The Venn diagram generated after the OTU clustering of effective tags from all samples with 97% consistency showed that the control group contained 831 OTUs, and the SeY group contained 886 OTUs, with 665 OTUs shared. In the control and SeY groups, there were 166 and 221 unique OTUs, respectively (Fig. 2B). All valid reads were classified taxonomically using QIIME (Supporting Information Table S1).

### Gut microflora of cecum

#### Richness and diversity of bacterial phylotypes

**Figure 3.**
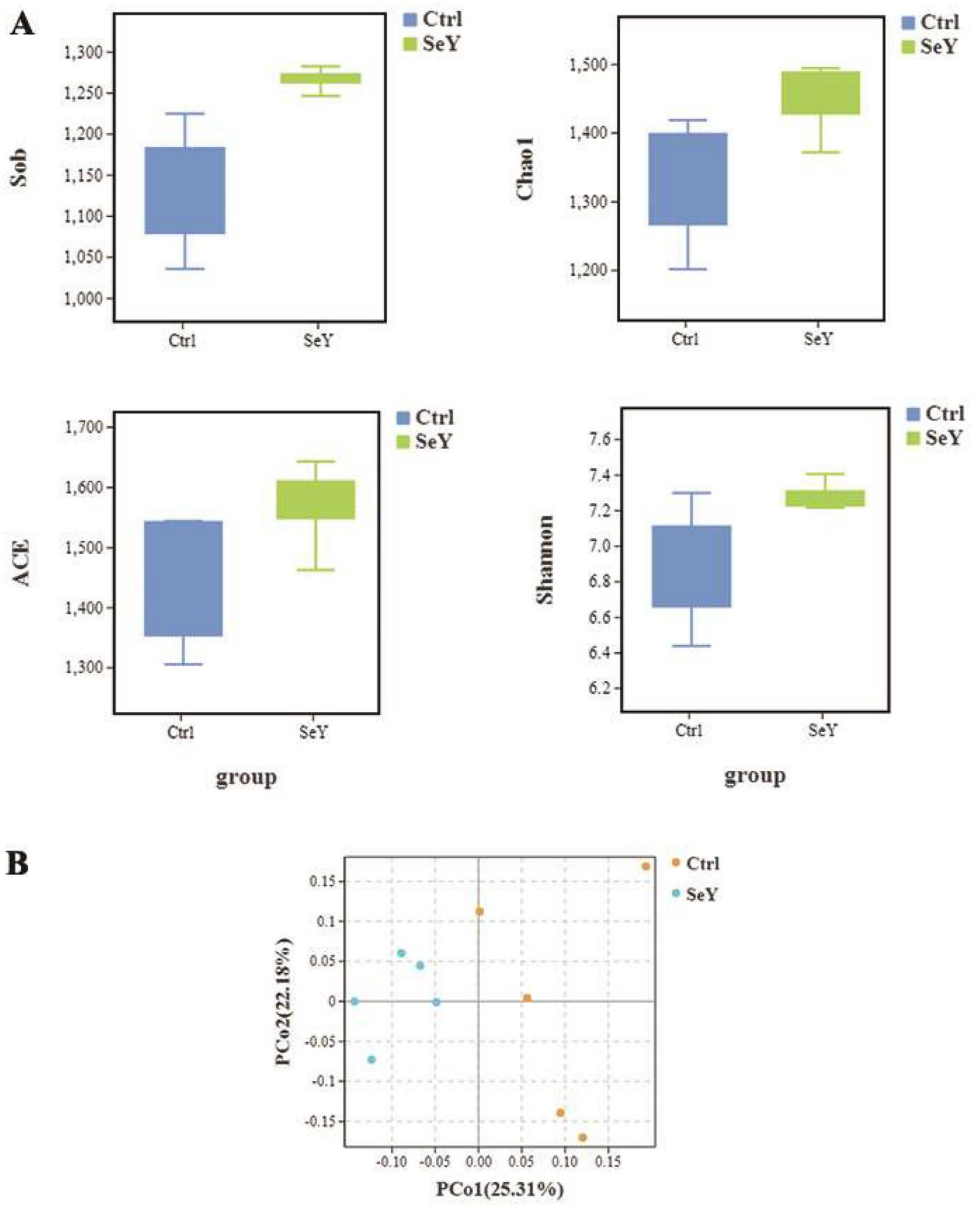
Effect of SeY on biodiversity of cecum microbiota of laying hens. (A) An alpha diversity index box graph was established based on the Sob, Chao1, ACE and Shannon indexes. (B) PCoA plot based on the weighted Unifrac metric.

In comparison to the control group diet, the SeY diet altered the alpha diversity index (*p <* .05; Fig. 3A). Unweighted UniFrac was used to calculate beta diversity, and principal coordinate analysis (PCoA) was used to measure the degree of similarity between microbial communities. As shown in Fig. 3B, there was increased similarity in the community structure in SeY group.

#### Bacterial taxonomic composition

**Figure 4.**
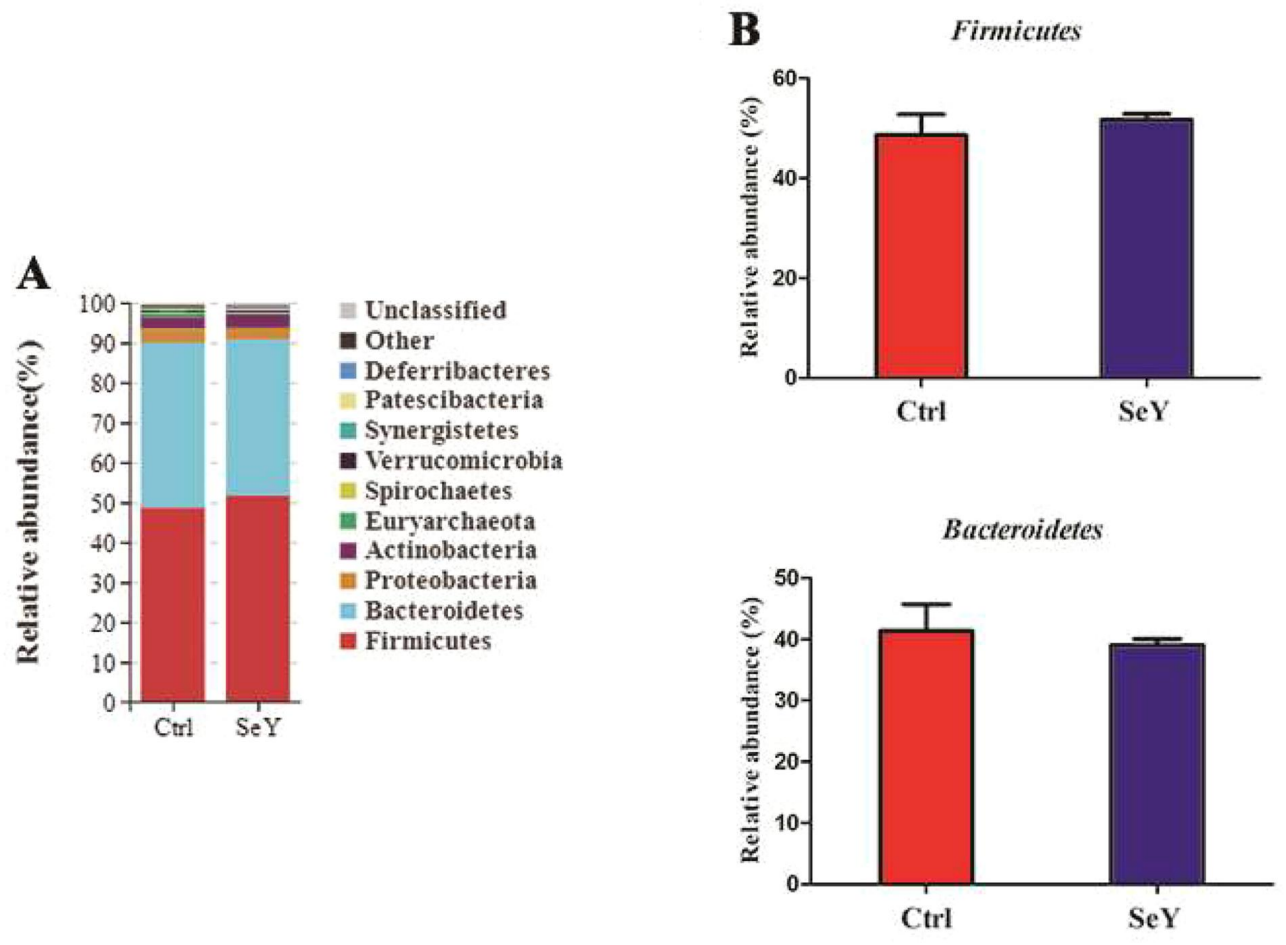
Relative abundance of the cecal microbiota at the phylum level in the Ctrl, and SeY treatments based on the 16S rDNA gene sequence. (A) The stack-column of the cecal microbiota from different groups at phylum level. (B) The relative abundance of *Firmicutes* and *Bacteroidetes* was expressed as mean ± SEM.

**Figure 5.**
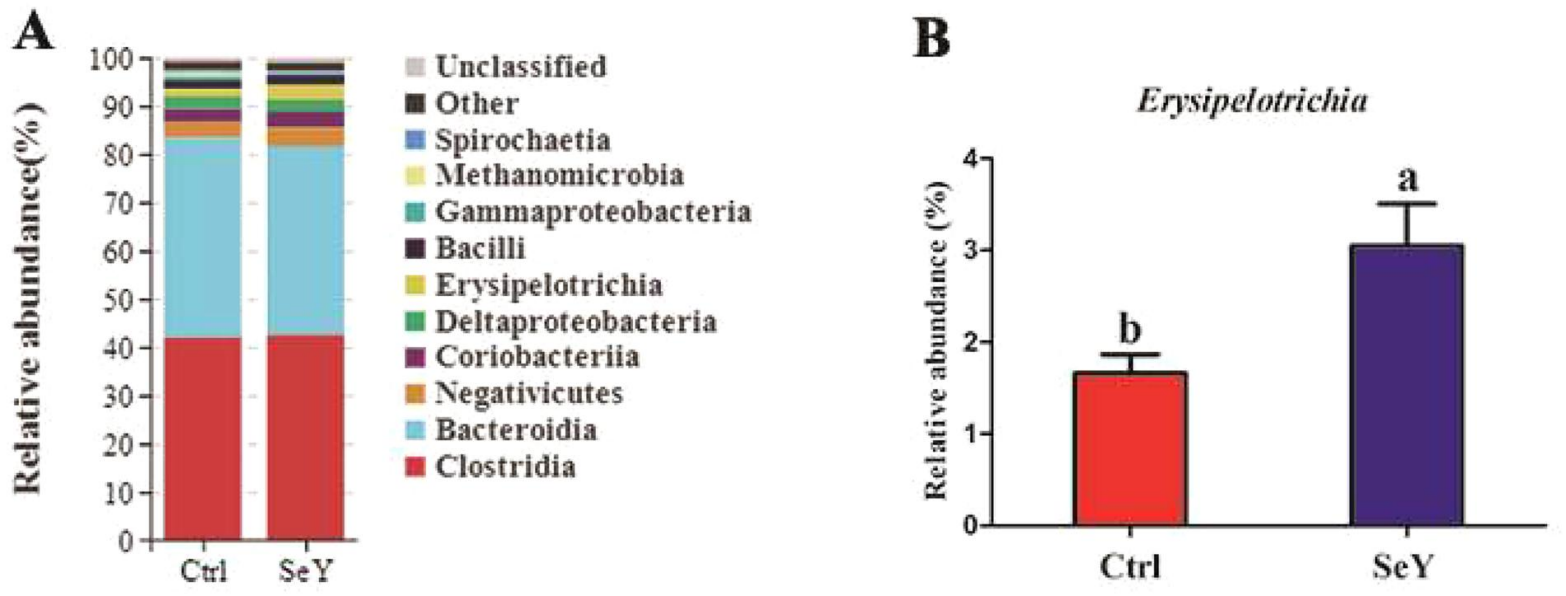
Relative abundance of the cecal microbiota at the class level in the Ctrl and SeY treatments based on the 16S rDNA gene sequence. (A) Bar graph the top 10 class from samples. (B) The relative abundance of Erysipelotrichia was expressed as mean ± SEM. Values on each bar with no common letter differ significantly (*p* < .05).

**Figure 6.**
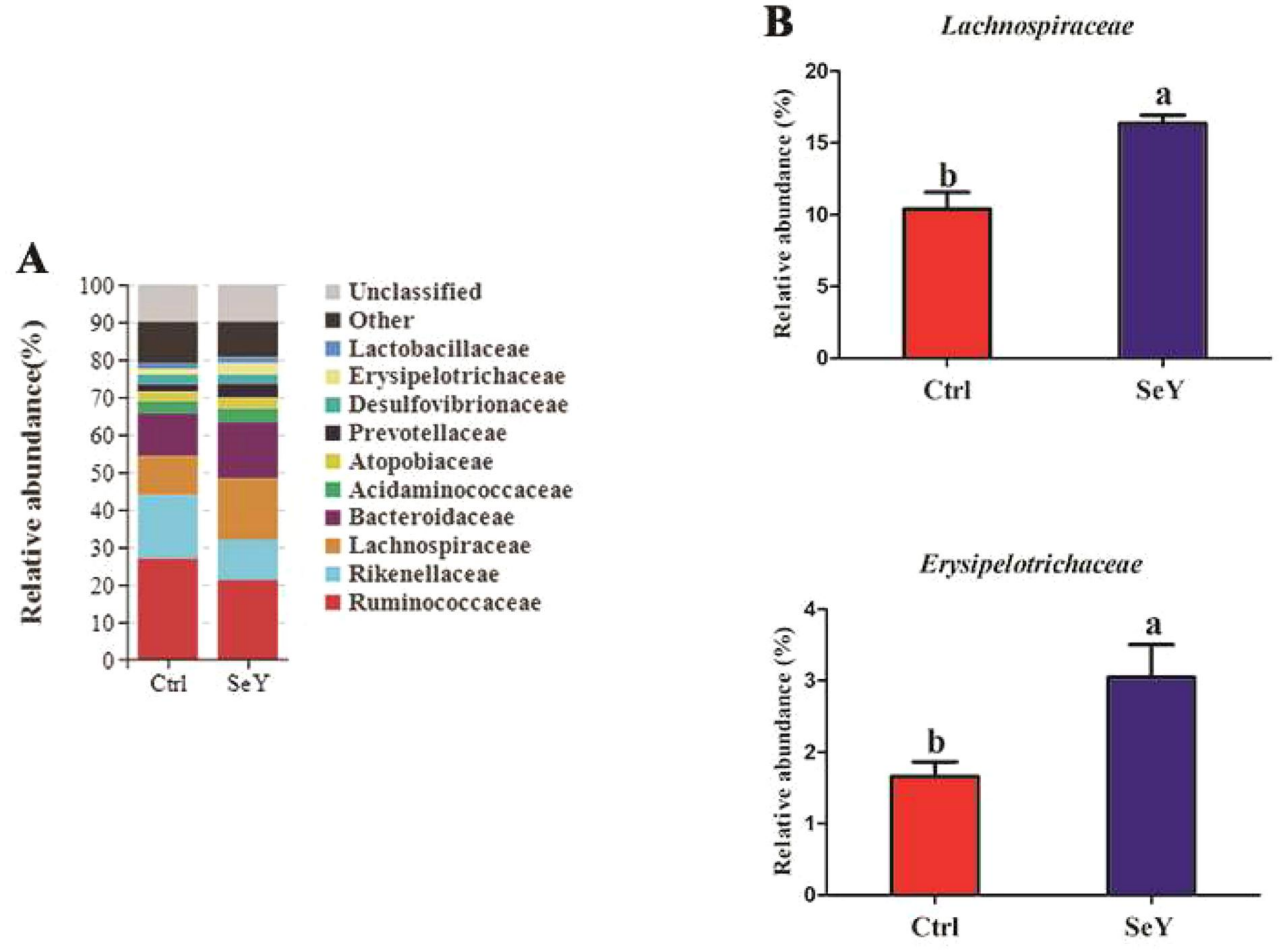
Relative abundance of the cecal microbiota at the family level in the Ctrl and SeY treatments based on the 16S rDNA gene sequence. (A) Relative abundance of the top 10 bacterial family were presented in each group. (B) The relative abundance of Lachnospiraceae and Erysipelotrichaceae were expressed as mean ± SEM. Values on each bar with no common letter differ significantly (*p* < .05).

**Figure 7.**
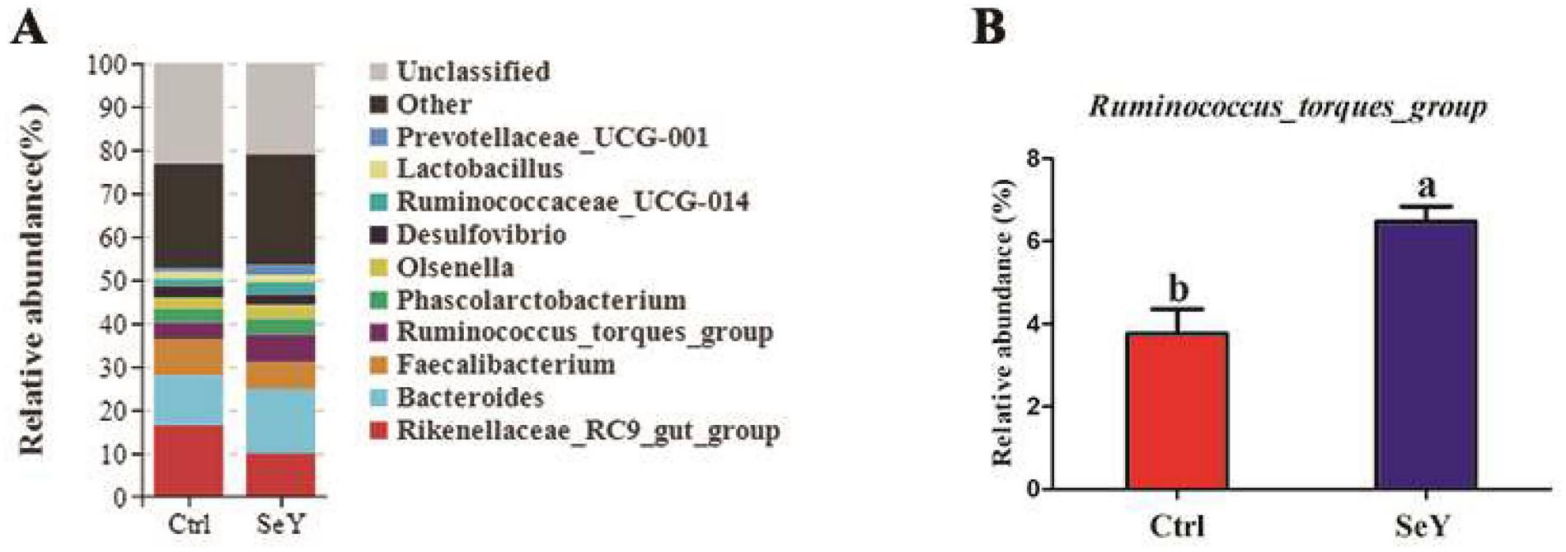
Relative abundance of the cecal microbiota at the genus level in the Ctrl and SeY treatments based on the 16S rDNA gene sequence. (A) The relative abundance of the top 10 genera from samples. (B) The relative abundance of Ruminococcus_torques_group was expressed as mean ± SEM. Values within a row with no common superscripts differ significantly (*p* < .05).

Furthermore, an in-depth analysis of the bacterial composition of the two groups showed the predominant phyla in all groups were *Firmicutes* and *Bacteroidetes* (Fig. 4A). However, there was not a significant difference between the control and SeY groups (*p >* .05; Fig. 4B). At the class level in the cecum of laying hens, Fig. 5A shows changes in relative abundance of species. An increased proportion of *Erysipelotrichia* was observed in the SeY group (*p <* .05; Fig. 5B). To illustrate specific changes in microbial taxa, the relative abundance of 10 predominant family was analyzed for each group (Fig. 6A). The results demonstrated that SeY enriched the abundance of *Lachnospiraceae* and *Erysipelotrichaceae* (*p <* .05; Fig. 6B). At the genus level, the top 10 genera and the bacterial taxa with a relative abundance >0.1% were presented in Supporting Information Table S2 and Fig. 7A, respectively. Compared to the control group, the relative abundance of *Ruminococcus_torques_group* was significantly changed in SeY group (*p <* .05; Fig. 7B).

#### Taxonomic characterization of the gut microbial profile

**Figure 8.**
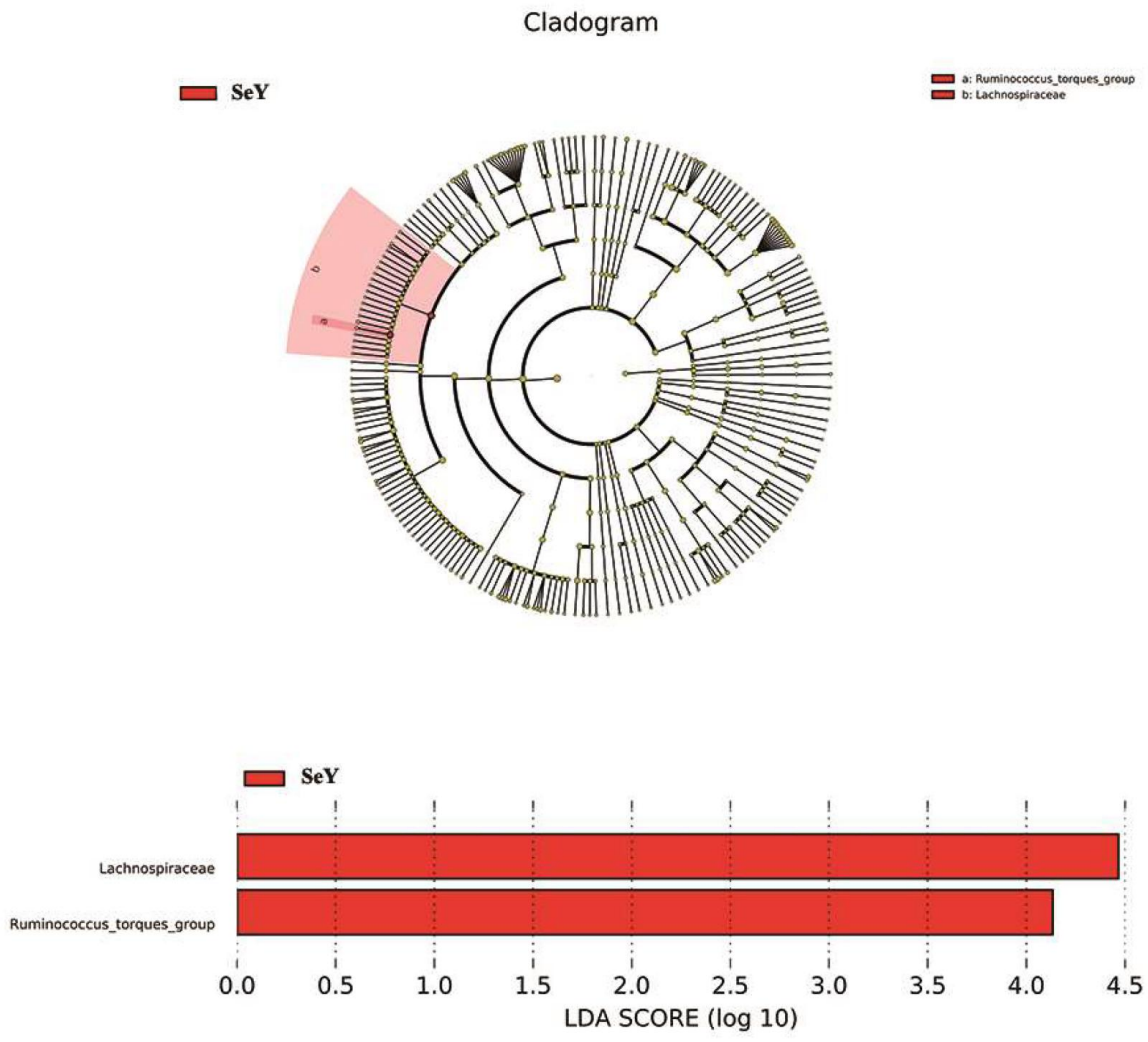
The main taxa of bacteria that were different in CT vs. SeY. (A) Cladogram of the main taxa of microbiota that were different on the basis of LEfSe analysis. (B) LEfSe analysis (taxa with LDA score > 4). color code: Red represents significantly different taxa, with their highest relative abundance in SeY.

LEfSe was used to analyze the detailed changes of intestinal microorganisms in laying hens fed dietary supplementation of SeY. Microbial contributions to the between-group differences among the groups were assessed using the LDA score. The main taxa that different between the two groups was the *Lachnospiraceae* and *Ruminococcus_torques_group* which were increased in SeY group (*p <* .05; Fig. 8).

#### Microbial potential functions

**Figure 9.**
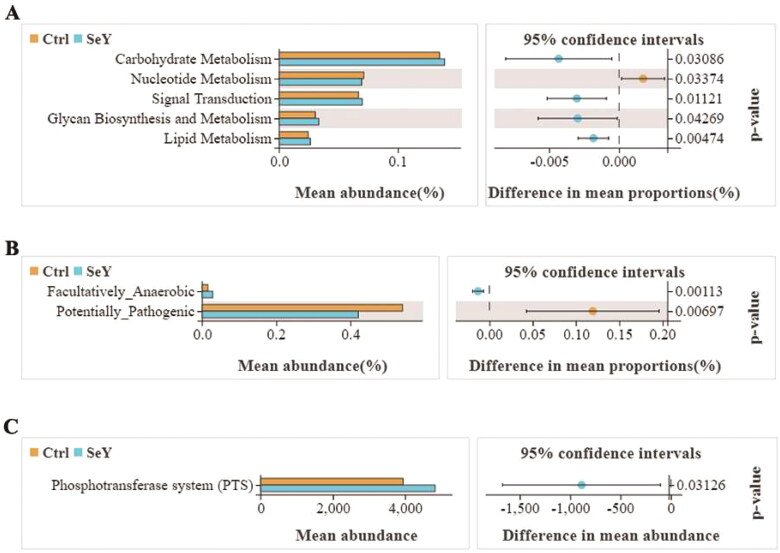
Functional divergence of gut microbiota across different dietary groups. (A) Tax4Fun functional profile of cecal microbiota communities under different treatments based on KEGG pathway analysis. (B) Bugbase predicted microbial community phenotypes and the corresponding bacterial contributions. Statistical significance was determined by Welch’s test. (C) Extended error bar plot for two-group analysis module comparison of PICRUSt predicted KEGG function data.

The gene functions such as carbohydrate metabolism, nucleotide metabolism, signal transduction, glycan biosynthesis and metabolism and lipid metabolism were detected in the two groups by the Tax4Fun method. Added SeY promoted carbohydrate metabolism, signal transduction, glycan biosynthesis and metabolism, and lipid metabolism (*p <* .05; Fig. 9A). We predicted phenotypes of gut microbiota through the BUGBASE software. The relative abundance of potentially_pathogenic phenotype presented a decreasing trend after adding SeY (*p <* .05; Fig. 9B). The functional profiles of microbial communities were determined using PICRUSt2, an updated version of a widely used metagenomic prediction tool. The pathway for phosphotransferase system was abundant in the SeY group (*p <* .05; Fig. 9C).

## Discussion

It is essential for animal growth that selenium is supplemented with additives to livestock and poultry diets to prevent selenium deficiency. There are several Se sources used in poultry diets, including sodium selenite, nano-Se, Se-Met, Se-Cys and SeY.^30^ To produce high quality eggs, Se sources with high biosafety and bioefficiency must be continuously explored and certified.

Optimal gastrointestinal health and function are key factors in improving feed efficiency, maintaining animal welfare and promoting sustainable animal nutrition.^31^ So far, a great deal of research on the application of selenium-enriched yeast in laying hens has focused on the production of selenium-enrich eggs.^32^ Although it has been extensively studied in laying performance, there are few reports on the effects of SeY on the intestinal health of laying hens.

The addition of SeY to the diet did not significantly affect the egg quality, and this is in accordance with the results observed in previous studies.^15^^,^^33^ Some reports, however, have demonstrated the positive effects of dietary organic Se supplementation on egg quality. Qiu et al. (2021) observed that SeY-fed laying hens produced smaller eggs than basal diet-fed laying hens during weeks 5–6 (*p <* .05).^34^ Meng et al. (2019) showed that supplementation of SeY (0.3 mg Se/kg of dry matter) to the diet significantly affected egg production and egg weight, also by improving feed conversion ratio (FCR) compared to that of the control group (*p* < .05).^35^ In our experiment, no beneficial or harmful influence on egg quality was found after the 21, 42 and 56d feeding trials. It may be due to differences in age, basal Se level, added Se level, genetic factors and storage methods that some of these results are inconsistent.

The intestinal tract is the main place where microorganisms live in the body, and it’s also the main organ for human and animal bodies to digest and absorb nutrients. To accurately evaluate the impact of nutritional interventions on important aspects like gut mucosa morphology and gut microflora composition, it is, therefore, important to monitor gastrointestinal function.^36^ The morphology of the intestinal villi determines the nutrient absorption capacity of the intestine. The longer the VH, the larger the absorption area of the intestine, and the stronger the absorption capacity of nutrients. An increase in CD indicates that the villi in the intestinal mucosa are atrophied and their absorptive capacity is decreased.^37^ It has been reported that SeY supplemented in basal diet could promote the development of liver and small intestine of chickens, and improve the microstructure of hepatocytes and mucosa of small intestine.^38^ Similarly, Read-Snyder et al. (2009) reported that longer and more narrow villi, greater surface perimeter, more shallow crypt depth and significantly greater VH/CD ratios in SeY-fed birds, compared with birds fed no supplemental Se or sodium selenite.^39^ In the current study, we observed that the Se supplementation did not have a significant effect on the intestinal morphology of laying hens. Our observations, however, seem to be inconsistent with previous findings. The apparent discrepancy may be due to the different forms and doses used in other studies. In high concentrations, Selenium can induce toxicity symptoms, which negatively impact laying hen performance and health. In summary, dietary SeY resulted in a minor alteration of cecal morphology, and consequently, the intestinal absorption capacity was not affected.

Gut microbiota are influenced by environmental and dietary factors and are associated with many physiological functions. The gut microbes play an important role in the digestion of nutrients and the extraction of energy. As a result, the gut microbiota profile can provide information regarding the host’s physiology status.^40^^,^^41^ SeY has many known physiologic effects, but its regulation of gut microbiota is unknown. Intestinal microbiota diversity plays a key role in colonization resistance against invading pathogens, and high diversity is associated with protection from foreign microorganisms.^42^ Using dietary supplementation with 100 mg/kg of SeY, we found that the cecal microbial diversity of laying hens was significantly increased. Our observation is in agreement with that of Diao (2020), who found that high-dose selenium yeast intervention could significantly improve the diversity and richness of intestinal flora in ApoE^-/-^ mice.^43^ In our study, *Firmicutes*, *Bacteroidetes* and *Proteobacteria* were the most abundant phyla identified in all experimental groups. Although the microbial communities were not significantly different at phylum, we found that *Firmicutes* and *Firmicutes*/*Bacteroidetes* ratio increased when the birds were supplemented with SeY. As a biomarker of gastrointestinal function, *Firmicutes*/*Bacteroidetes* ratio can indicate conditions of eubiosis.^44^ It has been suggested that yeast can be used as a good feed supplement for birds as it may act as a probiotic.^45^ In this research, we observed that at the genus level, SeY can increase the amount of *Ruminococcus_torques_group*. However, Lyra et al. (2010) demonstrated that *Ruminococcus_torques* was decreased in probiotics group compared with placebos, which revealed the pathogenicity of *Ruminococcus_torques* by damaging the mucosal barrier in IBS-D.^46^ The significant difference in the above results may be related to the species of probiotics and the different experimental animals. Also, we observed that SeY clearly increased *Erysipelotrichis* (class) and *Lachnospiraceae* (family) abundance. In agreement with our finding, it has been reported that *Lachnosirpraceae* increased to normal level after the intervention of SeY in ApoE−/− mice.^43^ At present, it has been demonstrated that *Lachnospiraceae* is considered to be intestinal probiotics, which is negatively correlated with intestinal inflammation.^47^ Consequently, *Ruminococcus_torques_group* and *Lachnospiraceae* may have an effect on immune systems of laying hens. The Tax4Fun gene function prediction revealed that the metabolism of cecal microbiota was significantly enriched in carbohydrate metabolism. The results showed that SeY improved carbohydrate metabolism of cecum contents in layers. Specifically, carbohydrate metabolism is primarily composed of tricarboxylic acid cycle, glycolysis and pentose phosphate pathway. According to the results of Chen et al. (2022), the optimum dietary Se activates glucose catabolic processes, fatty acid biosynthetic processes and energy production and hence produces higher liver lipid content.^48^ Similarly, the mean abundance of lipid metabolism was obviously increased relative to CT by SeY treatment. In another study, SeY could reduce cholesterol and alleviate gut disorders by regulating cholesterol metabolism-related genes and promoting cholesterol excretion, thereby improve dyslipidemia and further metabolic disorders caused by high-fat diet.^43^ Through the analysis of BugBase Feature prediction, the changes in cecal microbial function have been further verified. Previous research has indicated that SeY can significantly alleviate and prevent mycotoxins.^49^ Our findings demonstrated that the relative abundance of anaerobic and potentially_pathogenic phenotypes presented a decreasing trend after adding SeY, which reduced the intestinal health risk of laying hens. The analysis results of PICRUSt2 showed that the relative abundance of PTS was increased in SeY group. PTS is the most common sugar transport system in bacteria, which mainly participates in the transport of carbon sources and affects the biofilm formation and pathogenicity of pathogens.^50–52^ Therefore, we speculated that the addition of SeY improved metabolism health and maintained intestinal homeostasis by regulating the action of the PTS system.

In conclusion, dietary SeY supplementation had no effect on egg quality and intestinal morphology, but it significantly enriched microbial composition as shown by a higher ratio of beneficial bacteria and a lower ratio of harmful bacteria, promoting optimal gastrointestinal health in laying hens. The findings will provide further insight into how SeY acts in laying hens from the perspective of microbial mechanisms.

## Supplementary Material

Supplemental Material

Supplemental Material

## Data Availability

The datasets generated and analyzed during the study are available from the corresponding author on reasonable request.
