# Supplementary material for: Effects of selenium-enriched yeast dietary supplementation on egg quality, gut morphology and caecal microflora of laying hens
Source: Anim Biotechnol. 2024 Jan 9;35(1):2258188. doi: 10.1080/10495398.2023.2258188 (PMC12674293; doi:10.1080/10495398.2023.2258188)
Supplement: Supplemental Material [file LABT_A_2258188_SM4273.docx]

**Supplementary Table S2.** The relative abundance (>0.1%) of the bacterial at genus level of different samples.

| Genus | Ctrl-1 | Ctrl-2 | Ctrl-3 | Ctrl-4 | Ctrl-5 | SeY-1 | SeY-2 | SeY-3 | SeY-4 | SeY-5 |
| --- | --- | --- | --- | --- | --- | --- | --- | --- | --- | --- |
| Rikenellaceae_RC9_gut_group | 9.8394 | 25.7182 | 17.6509 | 17.5265 | 11.6456 | 10.931 | 12.0853 | 8.6258 | 11.9684 | 6.2287 |
| Bacteroides | 11.0447 | 13.4815 | 4.1311 | 13.2197 | 15.0072 | 15.2443 | 17.9765 | 13.7848 | 13.8458 | 13.3919 |
| Faecalibacterium | 11.8862 | 1.7821 | 10.0589 | 4.8672 | 14.1035 | 2.0348 | 7.7574 | 4.7596 | 2.9512 | 12.9369 |
| Ruminococcus_torques_group | 2.8328 | 3.9565 | 2.1724 | 5.5965 | 4.2589 | 7.456 | 5.8156 | 7.1439 | 6.386 | 5.5984 |
| Phascolarctobacterium | 2.6787 | 4.0449 | 2.1594 | 3.232 | 3.5251 | 3.3938 | 6.2599 | 0.4482 | 5.7709 | 2.1767 |
| Olsenella | 3.4123 | 2.1925 | 5.5297 | 1.0901 | 1.1665 | 5.5652 | 1.616 | 1.7166 | 4.8499 | 2.069 |
| Desulfovibrio | 2.1547 | 1.705 | 4.1634 | 2.71 | 1.785 | 1.8354 | 2.0603 | 2.2228 | 2.8481 | 2.9944 |
| Ruminococcaceae_UCG-014 | 0.3144 | 1.3559 | 0.6346 | 2.3491 | 3.8552 | 0.7312 | 1.9188 | 6.961 | 1.0063 | 3.9075 |
| Lactobacillus | 0.5672 | 0.6394 | 1.9975 | 3.8154 | 0.8684 | 3.2239 | 1.9286 | 0.994 | 1.817 | 0.4642 |
| Prevotellaceae_UCG-001 | 0.3175 | 1.0996 | 0.7414 | 0.9635 | 0.9229 | 1.1706 | 1.2441 | 3.3997 | 1.4401 | 4.59 |
| Ruminococcaceae_UCG-005 | 0.2928 | 1.5803 | 1.8875 | 3.0362 | 1.3876 | 1.7246 | 1.8003 | 1.7746 | 0.7822 | 1.0945 |
| Subdoligranulum | 4.781 | 0.823 | 2.3213 | 0.714 | 0.6409 | 1.5178 | 0.7998 | 0.8446 | 1.6569 | 0.9531 |
| Blautia | 1.2947 | 0.4195 | 4.2606 | 0.5719 | 1.2947 | 1.7911 | 0.9841 | 0.9848 | 1.7352 | 0.91 |
| Lachnoclostridium | 1.2083 | 0.6643 | 0.6928 | 1.094 | 1.221 | 1.3775 | 1.1783 | 1.8752 | 1.3618 | 1.8815 |
| Peptococcus | 0.3792 | 1.0838 | 1.6738 | 0.3762 | 0.6377 | 0.7386 | 0.8755 | 3.9729 | 2.2436 | 0.3751 |
| Erysipelatoclostridium | 0.746 | 0.9545 | 1.2141 | 0.5566 | 1.4357 | 1.7689 | 1.5567 | 0.6403 | 1.6249 | 1.1221 |
| Christensenellaceae_R-7_group | 1.5135 | 0.8706 | 1.7968 | 1.3358 | 1.0191 | 1.1854 | 0.7274 | 1.1434 | 0.9814 | 0.8301 |
| Parabacteroides | 1.6707 | 1.5236 | 0.6216 | 1.0326 | 1.1312 | 0.6056 | 1.3626 | 0.9025 | 0.6542 | 1.3435 |
| Shuttleworthia | 0.1572 | 0.3741 | 0.6961 | 0.6333 | 0.8364 | 0.6056 | 1.3856 | 1.6587 | 1.3369 | 2.315 |
| Ruminococcaceae_NK4A214_group | 0.8693 | 0.7142 | 1.5087 | 0.8406 | 1.0159 | 0.3988 | 0.4147 | 0.5336 | 0.5262 | 0.5503 |
| Butyricicoccus | 0.2312 | 0.8979 | 0.9389 | 0.3263 | 0.6025 | 0.3951 | 0.6879 | 1.4239 | 1.1663 | 0.661 |
| Oscillibacter | 0.0586 | 0.5374 | 1.3177 | 0.4721 | 0.7082 | 0.7201 | 0.543 | 0.3628 | 0.7858 | 0.5718 |
| Alistipes | 0.7768 | 0.4263 | 0.1554 | 0.3493 | 0.4871 | 0.4099 | 0.3094 | 0.7013 | 1.7956 | 0.5903 |
| Prevotellaceae_Ga6A1_group | 0.5579 | 0.9455 | 0.1004 | 1.1055 | 0.5929 | 0.1256 | 0.5398 | 0.2744 | 0.0676 | 0.5011 |
| Methanocorpusculum | 0.037 | 1.8819 | 0.0227 | 0.8214 | 1.1953 | 0.1477 | 0.2633 | 0.1189 | 0.2133 | 0.0369 |
| Barnesiella | 2.5986 | 0.3038 | 0.0907 | 0.2226 | 0.1955 | 0.1662 | 0.1481 | 0.1433 | 0.2418 | 0.2675 |
| Negativibacillus | 0.0401 | 0.1224 | 1.0554 | 0.2495 | 0.3749 | 0.3656 | 0.5496 | 0.1464 | 0.2773 | 1.0299 |
| Ruminococcaceae_UCG-008 | 0.1017 | 0.0884 | 0.0194 | 0.8752 | 0.4999 | 0.096 | 0.8162 | 0.5275 | 0.1564 | 0.7963 |
| Romboutsia | 0.6442 | 0.0317 | 0.6151 | 0.4184 | 0.1506 | 1.0377 | 0.2403 | 0.0793 | 0.4978 | 0.1445 |
| Eubacterium_coprostanoligenes_group | 0.2096 | 0.0453 | 0.2396 | 0.1996 | 0.3173 | 0.1477 | 0.5858 | 1.0702 | 0.3022 | 0.6825 |
| Akkermansia | 0.7336 | 0.2585 | 0.0648 | 0.3032 | 0.173 | 0.0739 | 0.1316 | 1.0245 | 0.3413 | 0.6302 |
| Ruminococcaceae_UCG-004 | 0.373 | 0.2925 | 0.2622 | 0.2226 | 0.2628 | 0.421 | 0.2501 | 0.7775 | 0.3627 | 0.3658 |
| Synergistes | 0.1695 | 0.7142 | 0.4403 | 0.5796 | 0.2275 | 0.1846 | 0.2008 | 0.2714 | 0.3662 | 0.206 |
| Eubacterium_hallii_group | 0.0555 | 0.0703 | 0.1133 | 0.499 | 0.2564 | 0.3397 | 0.3258 | 0.5702 | 0.4836 | 0.5626 |
| Odoribacter | 0.1449 | 0.5623 | 0.1133 | 0.3455 | 0.3974 | 0.2733 | 0.4871 | 0.3506 | 0.3556 | 0.1599 |
| Sellimonas | 0.1973 | 0.1882 | 0.1554 | 0.215 | 0.298 | 0.5909 | 0.1349 | 0.4391 | 0.5405 | 0.329 |
| Acinetobacter | 2.1239 | 0.1066 | 0.0065 | 0.1535 | 0.1795 | 0.0148 | 0 | 0.1616 | 0.0036 | 0.1875 |
| Eubacterium_brachy_group | 0.3699 | 1.2538 | 0.0939 | 0.1075 | 0.1218 | 0.2917 | 0.0757 | 0.1403 | 0.2489 | 0.0738 |
| Fournierella | 0.0524 | 0.1043 | 0.6961 | 0.1919 | 0.1474 | 0.1883 | 0.7504 | 0.0427 | 0.2702 | 0.0922 |
| Oscillospira | 0.7645 | 0.2653 | 0.0421 | 0.2956 | 0.1122 | 0.0923 | 0.0691 | 0.3567 | 0.0996 | 0.1937 |
| Erysipelotrichaceae_UCG-003 | 0.3576 | 0.059 | 0.0065 | 0.0269 | 0.0032 | 0.0369 | 0.0066 | 1.1983 | 0.0284 | 0.5595 |
| Oribacterium | 0.0339 | 0.1179 | 0.2137 | 0.119 | 0.2179 | 0.2696 | 0.2172 | 0.3689 | 0.384 | 0.2306 |
| GCA-900066575 | 0.0801 | 0.2517 | 0.136 | 0.215 | 0.1891 | 0.1699 | 0.2435 | 0.0823 | 0.2311 | 0.5042 |
| Faecalitalea | 0.4747 | 0.2313 | 0.0162 | 0.0691 | 0.0705 | 0.5872 | 0.1382 | 0.122 | 0.1564 | 0.0676 |
| CHKCI001 | 0.0154 | 0.0272 | 0.1166 | 0.2572 | 0.298 | 0.0332 | 0.5858 | 0.0671 | 0.0604 | 0.3259 |
| Ruminococcaceae_UCG-010 | 0.111 | 0.1247 | 0.2169 | 0.3071 | 0.1634 | 0.1588 | 0.1086 | 0.186 | 0.3378 | 0.0646 |
| Ruminiclostridium_5 | 0.111 | 0.2789 | 0.1684 | 0.1919 | 0.1538 | 0.096 | 0.1284 | 0.2348 | 0.1138 | 0.1414 |
| Mucispirillum | 0.0247 | 0.4376 | 0.1166 | 0.1382 | 0.0224 | 0.1366 | 0.1086 | 0.2561 | 0.128 | 0.2121 |
| Alloprevotella | 0.0277 | 0.2517 | 0.0453 | 0.1651 | 0.298 | 0.1219 | 0.3061 | 0.0305 | 0.1564 | 0.123 |
| Ruminococcaceae_UCG-013 | 0.3452 | 0.0839 | 0.1554 | 0.1036 | 0.1154 | 0.1625 | 0.1316 | 0.1982 | 0.1316 | 0.0922 |
| Faecalicoccus | 0.3791 | 0.1179 | 0.0291 | 0.0653 | 0.032 | 0.3951 | 0.079 | 0.0823 | 0.1244 | 0.0184 |
| Butyricimonas | 0.5888 | 0.4648 | 0.0032 | 0.1919 | 0.0224 | 0.0148 | 0.0066 | 0.0183 | 0.0107 | 0 |
| Parasutterella | 0.0493 | 0.3809 | 0.0259 | 0.119 | 0.1282 | 0.0739 | 0.2008 | 0.0549 | 0.0533 | 0.1506 |
| Candidatus_Stoquefichus | 0.0092 | 0 | 0.0227 | 0.0154 | 0.0256 | 0.9048 | 0.2172 | 0 | 0.0284 | 0.0123 |
| Treponema_2 | 0.0123 | 0.2675 | 0.0032 | 0.4146 | 0.0064 | 0.3176 | 0.0461 | 0 | 0.1138 | 0.0031 |
| Solobacterium | 0.0062 | 0 | 0 | 0.0038 | 0 | 0.3508 | 0.0099 | 0 | 0.7076 | 0.1045 |
| Enterococcus | 0.0154 | 0.0091 | 0.0097 | 0.0806 | 0.0513 | 0.6241 | 0.0099 | 0.0976 | 0.2382 | 0.04 |
| Family_XIII_AD3011_group | 0.1387 | 0.1292 | 0.136 | 0.0422 | 0.0481 | 0.0923 | 0.0362 | 0.1829 | 0.1885 | 0.083 |
| Tyzzerella | 0.4747 | 0.0023 | 0.0032 | 0.403 | 0.0385 | 0 | 0.0494 | 0.0671 | 0.0249 | 0.0031 |
| Eisenbergiella | 0.0185 | 0.0431 | 0.0227 | 0.0384 | 0.1314 | 0.0886 | 0.2534 | 0.0488 | 0.1636 | 0.1752 |
| Anaerofilum | 0.188 | 0.0703 | 0.1781 | 0.0691 | 0.0224 | 0.0037 | 0.1316 | 0.0335 | 0.0569 | 0.1107 |
| Erysipelotrichaceae_UCG-004 | 0.1079 | 0.0521 | 0.0194 | 0.0499 | 0.109 | 0.0111 | 0.181 | 0.2073 | 0.0676 | 0.0369 |
| Turicibacter | 0.1141 | 0.0204 | 0.0712 | 0.0844 | 0.0545 | 0.2474 | 0.0263 | 0.0244 | 0.1031 | 0.0369 |
| Megamonas | 0.0062 | 0.0113 | 0 | 0.023 | 0.1859 | 0.1366 | 0.1514 | 0.0274 | 0.2169 | 0.0123 |
| Eubacterium_nodatum_group | 0 | 0.0023 | 0 | 0.0154 | 0 | 0.4838 | 0 | 0.003 | 0.128 | 0.1076 |
| Kurthia | 0.0031 | 0 | 0 | 0.0154 | 0 | 0 | 0.5858 | 0 | 0 | 0 |
| Elusimicrobium | 0.0062 | 0.0181 | 0.123 | 0.0384 | 0.2179 | 0.0111 | 0.0263 | 0.064 | 0.0391 | 0.043 |
| GCA-900066225 | 0.0154 | 0.0227 | 0.0032 | 0.119 | 0.0449 | 0.0258 | 0.1448 | 0.0152 | 0.0356 | 0.1599 |
| Enterobacter | 0 | 0.093 | 0 | 0 | 0.1122 | 0.0037 | 0.0033 | 0.1982 | 0.0142 | 0.1599 |
| Megasphaera | 0 | 0.0136 | 0.0324 | 0 | 0.0673 | 0.0849 | 0.0132 | 0.0427 | 0.3058 | 0.0092 |
| Helicobacter | 0.0185 | 0.0068 | 0.0324 | 0.0537 | 0.1154 | 0.144 | 0.0033 | 0.1311 | 0.0107 | 0.0492 |
| Intestinimonas | 0.2497 | 0.0612 | 0.0389 | 0.0307 | 0.0224 | 0.0406 | 0.023 | 0.0335 | 0.0071 | 0.0154 |
| Sutterella | 0.111 | 0.0748 | 0.0097 | 0.0154 | 0.0385 | 0.0886 | 0.0987 | 0.0183 | 0.0071 | 0.0338 |
| Merdibacter | 0.0247 | 0.0181 | 0.0227 | 0.0653 | 0.0032 | 0.1514 | 0.0296 | 0.1098 | 0.0391 | 0.0215 |
| Aeriscardovia | 0.0031 | 0.0136 | 0.0032 | 0.0461 | 0.0128 | 0.2622 | 0.0856 | 0.0152 | 0.0071 | 0.0154 |
| Mailhella | 0.0062 | 0.1882 | 0.0324 | 0.0499 | 0.0128 | 0.0037 | 0.0724 | 0.0152 | 0.0178 | 0.0615 |
| Candidatus_Saccharimonas | 0.1356 | 0.0408 | 0.0162 | 0.0384 | 0.0385 | 0.0111 | 0.0099 | 0.0213 | 0.0249 | 0.1168 |
| Campylobacter | 0.0031 | 0.0567 | 0.0032 | 0 | 0.0705 | 0 | 0.0099 | 0.1677 | 0.0036 | 0.0154 |
| Escherichia-Shigella | 0.1665 | 0.0227 | 0.0097 | 0.0115 | 0.0256 | 0.0332 | 0.0033 | 0.0122 | 0.0036 | 0.0277 |
| Clostridium_innocuum_group | 0 | 0.0136 | 0.0162 | 0.0499 | 0.0192 | 0.1256 | 0.0066 | 0 | 0.0356 | 0.0061 |
| Streptococcus | 0 | 0.0113 | 0.0097 | 0.0077 | 0.032 | 0.0148 | 0.0033 | 0.1067 | 0.0462 | 0.0184 |
| Lachnospiraceae_UCG-010 | 0.111 | 0 | 0 | 0 | 0.0128 | 0 | 0.0033 | 0.0061 | 0 | 0.0031 |
