# Supplementary material for: Effects of selenium-enriched yeast dietary supplementation on egg quality, gut morphology and caecal microflora of laying hens
Source: Anim Biotechnol. 2024 Jan 9;35(1):2258188. doi: 10.1080/10495398.2023.2258188 (PMC12674293; doi:10.1080/10495398.2023.2258188)
Supplement: Supplemental Material [file LABT_A_2258188_SM4244.docx]

**Supplementary Table S1.** The tags and OTUs information of different samples based on the 16S rDNA gene sequence.

| Sample name | Raw Reads | Clean Reads | Raw Tags | Clean Tags | Effective Tags | Effective Ratio, % | OTUs |
| --- | --- | --- | --- | --- | --- | --- | --- |
| Ctrl-1 | 134670 | 132841 | 110712 | 108441 | 95913 | 71.22 | 1139 |
| Ctrl-2 | 133097 | 131275 | 106599 | 104754 | 94768 | 71.20 | 1079 |
| Ctrl-3 | 134381 | 132675 | 110120 | 108144 | 95941 | 71.39 | 1224 |
| Ctrl-4 | 121719 | 119760 | 96976 | 94912 | 83918 | 68.94 | 1182 |
| Ctrl-5 | 122895 | 121771 | 106199 | 103779 | 91877 | 75.08 | 1035 |
| SeY-1 | 127290 | 125453 | 103087 | 101181 | 89188 | 70.07 | 1194 |
| SeY-2 | 137372 | 135350 | 110737 | 108636 | 96108 | 69.96 | 1268 |
| SeY-3 | 128516 | 127043 | 106689 | 104775 | 93572 | 72.81 | 1246 |
| SeY-4 | 124321 | 132708 | 113149 | 110740 | 97303 | 72.44 | 1269 |
| SeY-5 | 136053 | 134525 | 114103 | 112026 | 99621 | 73.22 | 1282 |
